# The relevance of NMDA receptor antibody-specific index for diagnosis and prognosis in patients with anti-NMDA receptor encephalitis

**DOI:** 10.1038/s41598-023-38462-6

**Published:** 2023-08-04

**Authors:** Martin W. Hümmert, Konstantin F. Jendretzky, Karin Fricke, Marina Gingele, Dominica Ratuszny, Nora Möhn, Corinna Trebst, Thomas Skripuletz, Stefan Gingele, Kurt-Wolfram Sühs

**Affiliations:** Department of Neurology, Hannover Medical School, Carl-Neuberg-Str. 1, 30625 Hannover, Germany

**Keywords:** Neuroscience, Neurology

## Abstract

The clinical implications of the presence of anti-N-methyl-D-aspartate receptor (NMDAR)-specific intrathecal immunoglobulin G synthesis and whether it determines the diagnosis of anti-NMDAR encephalitis have not been thoroughly investigated yet. Thus, the aim of this study was to investigate whether the detection of intrathecal anti-NMDAR-specific IgG synthesis contributes to the diagnostic confirmation of anti-NMDAR encephalitis, to disease severity, and to prognosis in patients with positive serum anti-NMDAR-IgG. In this study, patients with detectable anti-NMDAR IgG in serum and/or cerebrospinal fluid (CSF) were included and separated into two groups that either met the 2016 criteria by Graus et al*.* of definite anti-NMDAR encephalitis (n = 27) or did not (n = 15). In a total, of 80 paired CSF/serum samples, antibody titers were titrated manually and end-point titer levels were carefully determined in a blinded manner to the subgroup attribution. The disease course was assessed via the modified Rankin Scale (mRS) and prognosis was estimated by the anti-NMDAR Encephalitis One-Year Functional Status (NEOS) score. With respect to whether the diagnostic Graus criteria for definite anti-NMDAR encephalitis were fulfilled, a significantly unequal distribution of intrathecal anti-NMDAR antibody-specific synthesis could be shown with a high negative predictive value in case of a negative anti-NMDAR antibody-specific index (NMDAR AI, p = .008. OR = 23.9, sensitivity = 1.0, specificity = 0.4, negative predictive value = 1). A weak correlation was found between the CSF antibody titer and mRS value at the time of sample collection (*r*_*s*_ = .37, p = .008, 95% CI [.09, .59]). During the disease course a higher delta-mRS value formed of the mRS at initial presentation minus that at the last recorded presentation correlated with a higher NMDAR AI at first lumbar puncture (*r*_*s*_ = − .56, p = .017, 95% CI [− .83, − .11]). No association with the prognostic NEOS score was found. In conclusion, a negative antibody-specific index for anti-NMDAR IgG antibodies has a highly negative predictive value for the diagnosis of anti-NMDAR encephalitis. Yet, a positive NMDAR AI alone does not allow the diagnosis of anti-NMDAR encephalitis.

## Introduction

Since the first description of anti-N-methyl-d-aspartate receptor (NMDAR) encephalitis, the most common antibody-mediated encephalitis known so far, previously unclassifiable encephalitides have experienced increasing attention and therapeutic options^1,2^. An integral part of the diagnosis is the examination of the cerebrospinal fluid (CSF), which often reveals inflammatory changes^3^. It has been shown that the presence of quantitative intrathecal immunoglobulin synthesis is characteristic of anti-NMDAR encephalitis and associated with clinical severity^4^. Moreover, growing evidence implicates that the correct diagnosis of anti-NMDAR encephalitis commonly requires the detection of anti-NMDAR immunoglobulin G (IgG) in CSF^5–7^. As anti-NMDAR-IgG itself pathologically modulates the neuronal cell surface^8^, a sufficient concentration in the central nervous system seems to be required for the clinical manifestation of anti-NMDAR encephalitis. The exact clinical implications of the presence of anti-NMDAR-specific intrathecal IgG synthesis and whether it determines the diagnosis of anti-NMDAR encephalitis have not been thoroughly investigated to date. The aim of this study was to investigate in serum and/or CSF whether the detection of intrathecal anti-NMDAR-specific IgG synthesis contributes to the diagnostic confirmation of anti-NMDAR encephalitis in patients with positive serum anti-NMDAR-IgG, and whether the anti-NMDAR-IgG titer and/or antibody-specific index level allows an estimation of disease severity and functional outcome.

## Patients and methods

### Patient characteristics

Medical records of patients admitted to the Department of Neurology at Hannover Medical School between 2009 and 2021 were screened for detection of anti-NMDAR immunoglobulin G (NMDAR-IgG).

Patients were included when either anti-NMDAR antibodies were detectable in serum and/or CSF. All patients who fulfilled the criteria of *definite anti-NMDAR encephalitis*, according to the 2016 review by Graus et al*.*^9^, were allocated to subgroup one. Thus, after exclusion of other diseases at least one typical symptom for anti-NMDAR encephalitis had to be present (abnormal behavior/cognitive dysfunction; speech dysfunction; seizures; movement disorder/dyskenias/rigidity; decreased level of consciousness; autonomic dysfunction or central hypoventilation). Furthermore, at least one abnormal paraclinical examination result was required (electroencephalography (EEG), CSF pleocytosis, and/or positive oligoclonal band (OCB) finding). A second subgroup was formed out of all patients, who did not meet the criteria of *definite anti-NMDAR encephalitis*, according to the 2016 review by Graus et al*.*^9^ This second subgroup was referred to as "anti-NMDAR-antibody only" (NAO). From 42 patients paired CSF/serum samples were collected. From 11 of these patients, follow-up CSF/serum samples were available of at least two time points. Including these follow-up samples (for the calculation of delta antibody-specific index) 80 paired CSF/serum samples were analyzed. The modified Rankin Scale (mRS), reflecting the extent of functional impairment due to neurological disease, and the anti-NMDAR Encephalitis One-Year Functional Status (NEOS) score, a prognostic outcome measure for functional status one year after diagnosis, were extracted from the medical records for all patients^10,11^. The NEOS score was determined for the time of hospital admission. The peak mRS score during the hospital stay, reflecting the most pronounced clinical affection during the disease course, and the mRS score at discharge were evaluated. In addition, clinical parameters including the need for and duration of intensive care, abnormalities on 1.5 or 3.0 T magnetic resonance imaging (MRI) and/or EEG, and evidence of ovarian teratoma were captured. Focal or diffuse slowing and signs of increased cerebral excitability were, for example, considered as abnormal EEG results. MRI abnormalities consisted of hyperintense lesions in the T2-weighted fluid-attenuated inversion recovery sequences that involved the grey or white matter and were consistent with demyelination or inflammation.

### Standard protocol approvals, registrations, and patient consents

This study was approved by the institutional ethics committee of the Hannover Medical School (no. 8172-BO-K-2018).

### Sample collection and preanalytical preparation

Cerebrospinal fluid was obtained by lumbar puncture and immediately analyzed along with the respective paired serum sample in the neurochemistry laboratory of the Department of Neurology, as reported previously^12,13^. Briefly, the CSF cell count was determined using the Fuchs-Rosenthal chamber. After centrifugation, the cell-free supernatant was used for further diagnostics. Albumin and IgG in serum and CSF were measured by nephelometry (Beckman Coulter IMMAGE). For determination of the blood–CSF barrier function, the age-adjusted upper limit of albumin quotient (QAlb) was used by applying the formula QAlb = 4 + (age in years/15) × 10^3,14^. Quantitative measurements of the intrathecal IgG production were determined by calculating the CSF / serum ratios for IgG (QIgG) using the formula QIgG = CSF IgG total [mg/l] / serum IgG total [g/l]. The upper limits of the reference ranges, denoted as Qlim(IgG), were computed relative to QAlb using Reiber's revised hyperbolic functions (Qlim[IgG] = 0,93 × [(QAlb)^2^ + 6 × 10^–6^]^½^ – 1,7 × 10^–3^). Values of QIgG greater than Qlim(IgG) were interpreted as indicative of (unspecified) intrathecal immunoglobulin synthesis^14^. CSF-specific oligoclonal bands were assessed by isoelectric focusing in polyacrylamide gels with consecutive silver staining^15^. BIOCHIPS (EUROIMMUN Medizinische Labordiagnostika AG, Lübeck, Germany) of human embryonic kidney cells (HEK 293) employing recombinant NR1 subunit of the NMDA receptor were used to detect anti-NMDAR-IgG. After incubation with patients’ serum (primary dilution 1:10) or CSF (primarily undiluted, 1:1), indirect immunofluorescence was performed and positive immunofluorescence was determined by an experienced rater using a 400 × quantification with the EUROStar III Plus microscope (EUROIMMUN Medizinische Labordiagnostika AG, Lübeck, Germany). The titer level was determined as the end-point titer as the highest dilution for which a positive staining signal could still be determined.

The determination of the intrathecally formed specific fraction of anti-NMDAR antibodies (Q_spec_) was performed according to Reiber and Lange^16^. Thus, the calculation of the NMDAR-IgG CSF/serum antibody-specific index (AI) was performed according to the formula Q_spec_/Q_IgG_ ([CSF NMDAR-IgG / serum NMDAR-IgG] / [CSF IgG total / serum IgG total]). In case of intrathecal IgG production (Q_IgG_ > Q_lim_), as indicated by the Reiber diagram, the AI was calculated as Q_spec_/Q_lim_. As previously used in other cohorts a restrictive cut-off value for a positive AI was set to values > 2 based on the titer scale^17–19^.

### Statistics

Results were analyzed with GraphPad Prism 5.02 (GraphPad Software, USA). The D'Agostino & Pearson omnibus normality test was used for the determination of a Gaussian distribution. Results were tested for significance using the Kruskal–Wallis test with Dunn multiple comparison post hoc test for group comparison for three or more groups in nonparametric study samples. For comparison of two groups in nonparametric study samples the Mann–Whitney-U test was performed. One-way analysis of variance with Tukey's Multiple Comparison Test was used for group comparison in study samples with normal distribution. Spearmen's rho was used to correlate two continuous variables in nonparametric study samples. Fisher´s exact test was used to measure the independence of two categorical variables. To calculate the suitability of the AI as a diagnostic parameter, a receiver operating characteristic (ROC) analysis was performed with calculation of an area under the curve (AUC). The Youden Index was used to determine the best AI cut-off. P-values < 0.05 were considered statistically significant.

## Results

### Anti-NMDAR encephalitis subgroups

In total, a study population of 42 patients with IgG-type anti-NMDAR antibodies was investigated (Table 1). Twenty-seven of all 42 patients (64%) met the criteria for *definite anti-NMDAR encephalitis*. The remaining 15 patients (36%) who did not fulfill the described criteria were included in the NAO subgroup. In ten of these cases (10/15), another competing diagnosis was present: Seven had another inflammatory disease such as multiple sclerosis, three patients had another structural brain disease such as a brain tumor or intracranial bleeding. The remaining five cases showed no clinical signs of encephalitis.

**Table 1:** Patient characteristics.

	All patients (n = 42)	*Definite anti-NMDAR encephalitis* (n = 27)	*Anti-NMDAR-antibody only* (n = 15)
Age in years, *median* *(IQR 25%–IQR 75%)*	34 (21–40)	26 (21–39)	36 (23–49)
Sex: female, n (%)	30 (71)	21 (78)	9 (60)
MRI findings, n (%)	15 (38)^1a^	13 (50)^1b^	2 (15)^1c^
EEG findings, n (%)	21 (70)^2a^	19 (79)^2b^	3 (50)^2c^
Teratoma, n (%)	5 (15)^3a^	5 (20)^3b^	0 (0)^3c^
CSF pleocytosis, n (%)	20 (51)^4a^	13 (54)^4b^	7 (47)
Positive OCB (Type 2 or 3), n (%)	23 (61)^5a^	17 (71)^5b^	6 (43)^5c^
mRS at presentation, *median* *(IQR 25%–IQR 75%)*	3 (2–5)	3 (2–5)	3 (2–3)
NEOS score, *median* (*IQR 25%–IQR 75%*)	2 (1–3)^6a^	2 (1–3)^6b^	2 (1–3)^6c^

Data of patients missing: 3^1a^, 12^2a^, 8^3a^, 3^4a^, 4^5a^, 9^6a^, 1^1b^, 3^2b^, 2^3b^, 3^4b^, 3^5b^, 5^6b^, 2^1c^, 9^2c^, 6^3c^, 1^5c^, 4^6c^.

### Patient characteristics

The median age of all patients was 34 years (interquartile range from 25 to 75%: 21–40) and 71% (n = 30/42) were women. Regarding the examination results at admission, 21/30 patients (70%) showed abnormal EEG findings. Fifteen patients (38%, n = 15/39) had abnormal MRI findings. A teratoma was found in five patients (15%, n = 5/34). Twenty patients (51% n = 20/39) had an elevated CSF cell count (≥ 5 cells/µl) and 23 patients (61%, n = 23/38) had the finding of CSF-specific oligoclonal bands (type 2 or 3, Table 1). There was no significant difference in the mean CSF cell count between the groups *definite anti-NMDAR encephalitis* and NAO (mean of the group of all patients: 20 cells/µl; mean of the group with *definite anti-NMDAR encephalitis:* 22 cells/µl; mean of the group with NAO: 16 cells/µl).

### Comparison of anti-NMDAR antibody titers in serum and CSF and level of NMDAR AI between subgroups

**Figure 1:**
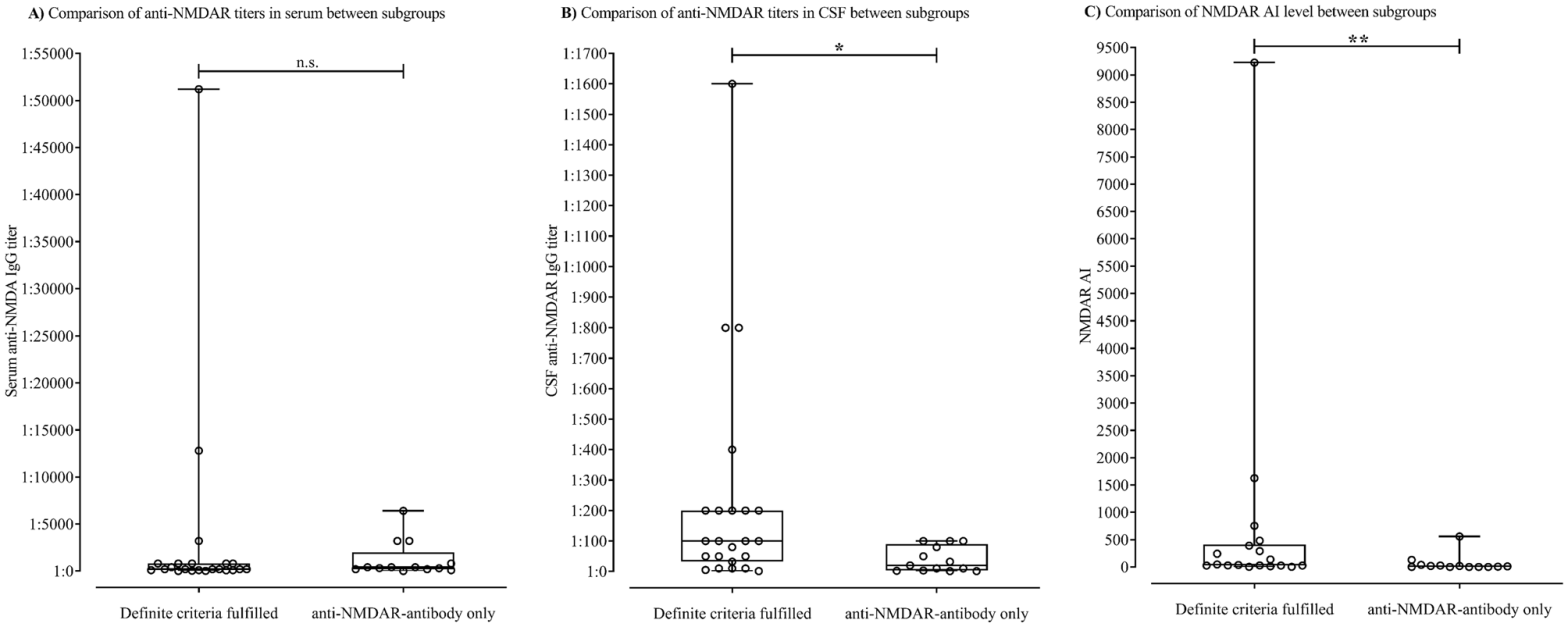
Comparison of anti-NMDAR titers in serum and CSF and level of NMDAR AI between subgroups. (**A**) Comparison of anti-NMDAR titers in serum between subgroups. (**B**) Comparison of anti-NMDAR titers in CSF between subgroups. Significant difference between subgroups *definite anti-NMDAR encephalitis* and *NAO* (p < .05). (**C**) Comparison of NMDAR AI level between subgroups. Significant difference between subgroups *definite anti-NMDAR encephalitis* and *NAO* (p < .05). The circles represent the level of respective patient result at initial presentation. The box represents the first quartile to the third quartile of the data (interquartile range). The vertical line within the box represents the median. Lower and upper whiskers represent the lower 25% of scores outside the middle and the upper 25% of scores outside the middle. The lines at the end of the whiskers represent the minimum and the maximum values of the data. AI, antibody-specific index; NAO, anti-NMDAR-antibody only; n.s., not significant.

A comparison between the subgroups in terms of anti-NMDAR IgG levels in serum and CSF, and the level of the NMDAR AI showed a significant difference regarding the level of CSF titer in both subgroups of patients fulfilling the criteria for definite anti-NMDAR encephalitis and patients of the NAO group (p < 0.05, Fig. 1B). Further differences were found between the subgroups when comparing the level of NMDAR AI (p < 0.05, Fig. 1C). Serum titers for anti-NMDAR IgG did not differ significantly between patients with definite anti-NMDAR encephalitis and patients who did not meet the criteria of definite encephalitis (p = 0.39, Fig. 1A).

### Evaluation of NMDAR AI as a diagnostic biomarker

Categorical groups were defined with respect to (a) the presence of a positive or negative NMDAR AI finding, and (b) whether the diagnostic Graus criteria for *definite anti-NMDAR encephalitis* were met or not met. A significant difference between the two described groups was found when applying the Fisher's exact test. Thus, a significantly unequal distribution could be shown with regard to the presence of a positive NMDAR AI and the simultaneous presence of the fulfilled diagnostic criteria with a high negative predictive value (p = 0.008. OR = 23.9, sensitivity = 1.0, specificity = 0.38, negative predictive value = 1; Table 2). The ROC analysis of the AI resulted in an area under the curve (AUC) of 0.79 (95% CI 0.63 to 0.96, p = 0.0057; Supplemental Fig. 1). Using the Youden index, the best discriminatory cut-off was an AI of 22: When testing a different diagnostic cut-off for NMDAR AI with values of 4 and 22, a significantly unequal distribution was shown for an NMDAR AI cut-off of 22 (p = 0.008. OR = 11.3, sensitivity = 0.83, specificity = 0.69, negative predictive value = 0.75). Using a value of 4, no significantly unequal distribution was found (Table 2). All following analyses were performed with the 27 patients who met the criteria of *definite anti-NMDA receptor encephalitis*.

**Table 2:** Contingency table for patients fulfilling or not fulfilling definite anti-NMDAR encephalitis criteria depending on NMDAR (AI) value.

n = 31	Fulfilling definite criteria	Not fulfilling definite criteria	p (OR; sensitivity; specificity; negative predictive value)
AI > 2	18	8	.008 (23.9; 1.0; 0.38; 1.0)
AI ≤ 2	0	5	
AI > 4	17	8	ns (10.6; 0.94; 0.38; 0.83)
AI ≤ 4	1	5	
AI > 22	15	4	.008 (11.3; 0.83; 0.69; 0.75)
AI ≤ 22	3	9	

### Correlation of anti-NMDAR CSF titer and NMDAR AI level with disease severity

**Figure 2:**
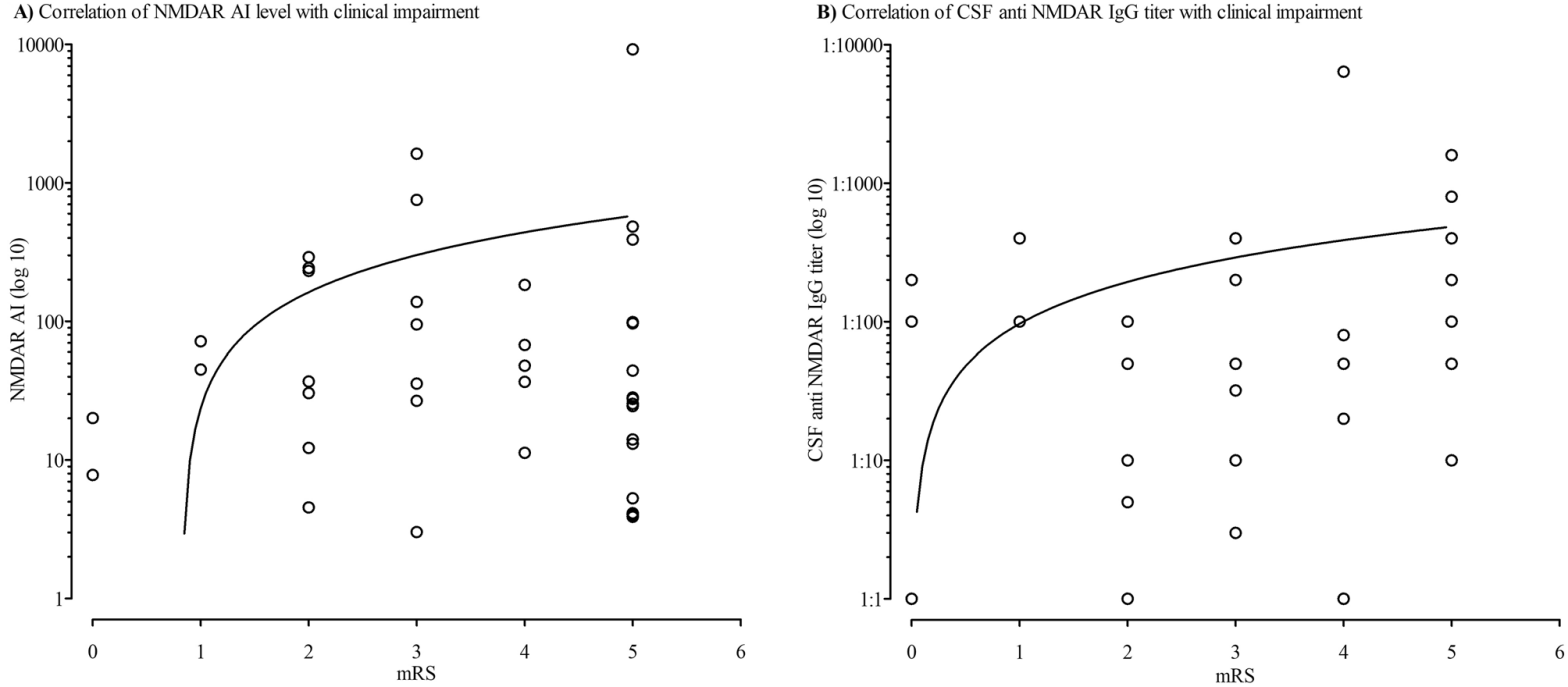
Correlation of anti-NMDAR CSF titer and NMDAR AI level with clinical impairment measured by mRS. (**A**) Correlation of NMDAR AI level with clinical impairment. (**B**) Correlation of CSF anti NMDAR IgG titer with clinical impairment. Significant correlation between CSF anti NMDAR IgG titer and mRS (*r*_s_ = .37, p = .008, 95% CI [.09, .59]). Y axis formatted in log 10 for better overview. The circles represent the level of respective individual patient result at each determination with the corresponding mRS value at the same time. AI, antibody-specific index; CSF, cerebrospinal fluid; mRS, modified rankin scale.

To investigate the association between anti-NMDAR IgG antibody titer or NMDAR AI values and disease severity, the results of all lumbar punctures, including follow-up results, were correlated with the concurrent mRS findings. There was no correlation between the NMDAR AI level and patients´ respective clinical impairment measured by mRS (*r*_*s*_ = − 0.09, p = 0.579, 95% CI [− 0.4, 0.24]). A weak correlation was found between the CSF anti-NMDAR IgG antibody titer and mRS value at the time of sample collection (*r*_s_ = 0.37, p = 0.008, 95% CI [0.09, 0.59]; Fig. 2B).

### Correlation of NMDAR AI level with clinical course

**Figure 3:**
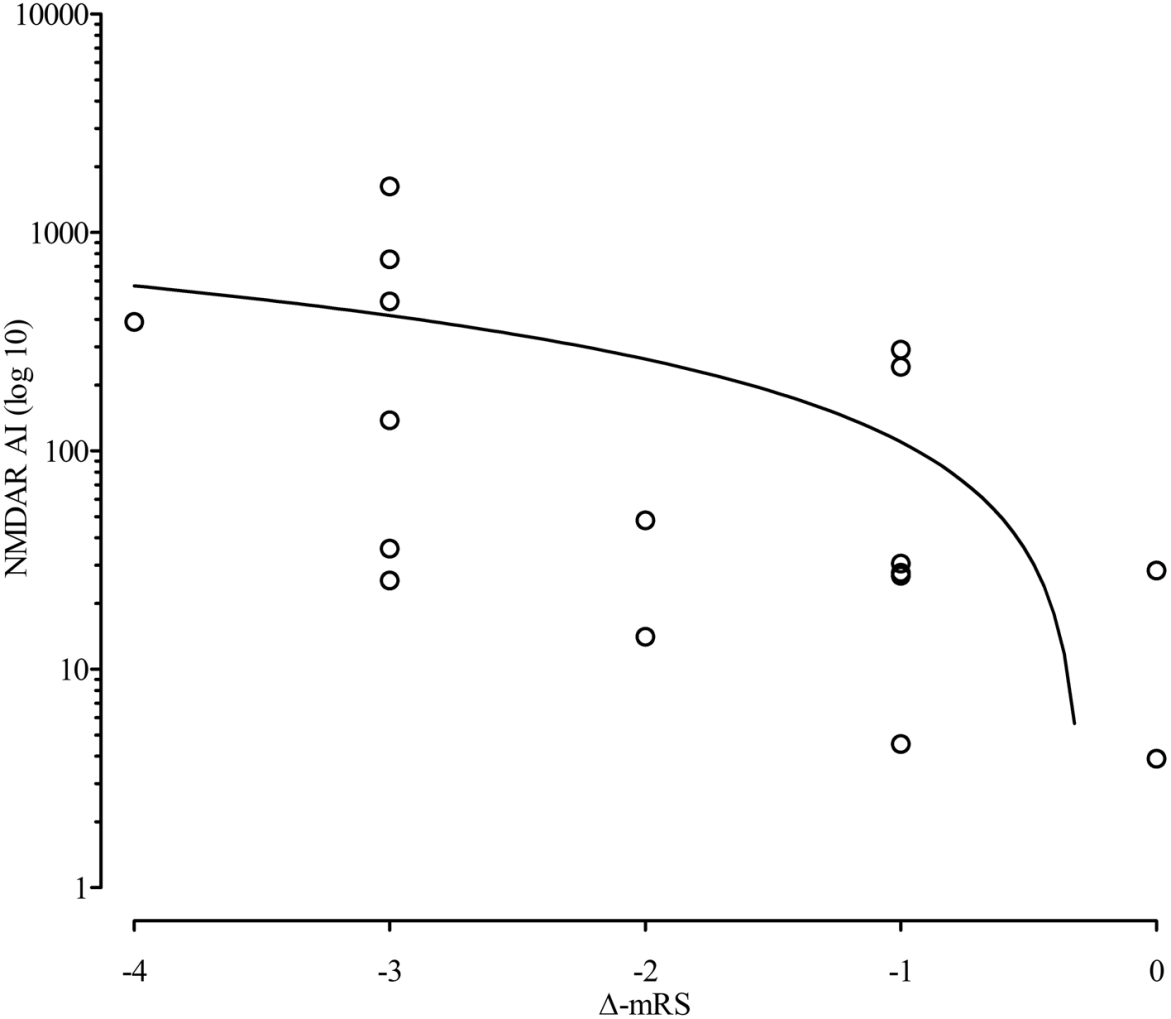
Correlation of NMDAR AI level with delta mRS. Significant correlation of NMDAR AI level with delta mRS (*r*_s_ = − .56, p = .017, 95% CI [− .83, − .11]). The circles represent the level of initial NMDAR AI with respective individual delta mRS. Delta mRS was composed of the mRS of the initial presentation minus the mRS of the last recorded presentation. AI, antibody-specific index; mRS, modified rankin scale.

**Figure 4:**
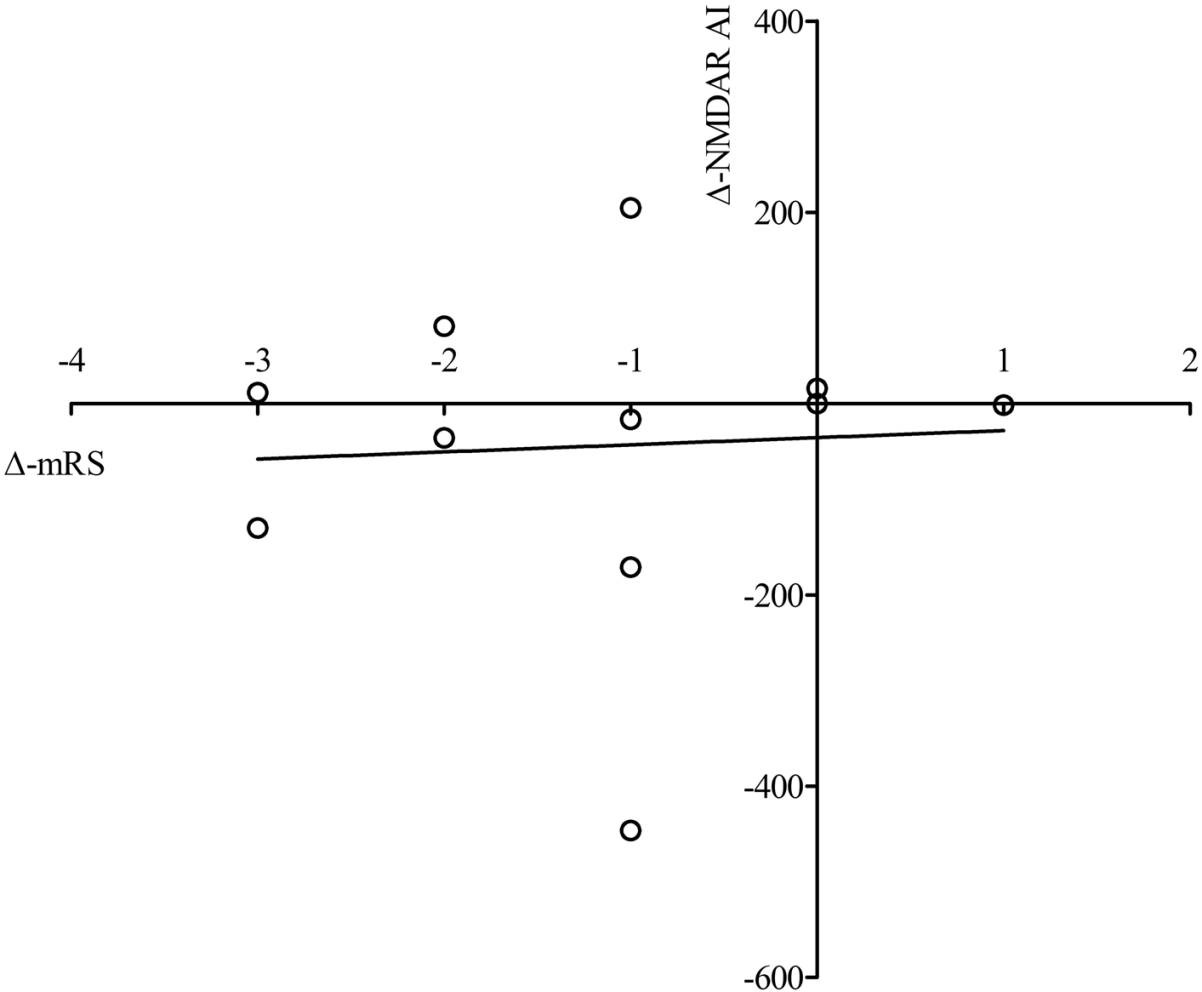
Correlation of delta NMDAR AI level with delta mRS. Circles represent level of delta AI with respective individual delta mRS. Delta values were composed of the AI and corresponding mRS of the initial LP minus that of the last puncture. AI, antibody index; mRS, modified rankin scale.

To compare the level of NMDAR AI with the clinical course of 17 patients with a median follow-up of 15 months, a delta-mRS value was formed, which consisted of the mRS at initial presentation minus that of at last recorded presentation (Fig. 3). There was a correlation between delta mRS and NMDAR AI at first lumbar puncture (*r*_s_ = − 0.56, p = 0.017, 95% CI [− 0.83, − 0.11]). In 11 patients who underwent at least two lumbar punctures and had a detectable anti-NMDAR antibody titer in serum and CSF with a median follow-up duration of 19 months, a delta NMDAR AI was formed (Fig. 4). No correlation could be detected when evaluating delta mRS (mRS at first lumbar puncture minus mRS at last lumbar puncture) vs. delta AI (AI at first lumbar puncture minus AI at last lumbar puncture; *r*_s_ = 0.1, p = 0.75, 95% CI [− 0.54, 0.67]). To estimate if an unfavorable outcome was related to a high NMDAR AI, the NEOS score was retrospectively determined for initial patient admission. The NEOS score and the magnitude of the NMDAR AI showed no relevant correlation (*r*_s_ = − 0.28, p = 0.34, 95% CI [− 0.71, 0.32]).

## Discussion

This study provides a comprehensive analysis of the (AI) in patients with anti-NMDAR-IgG antibodies. The AI has proven to be a useful tool to diagnose infections of the central nervous system, by demonstrating pathogen-specific antibody synthesis in the CNS, particularly in circumstances when PCR tests have a low positivity rate e. g. due to rapid viral clearance or during later stages^20^. In comparison to mere serum and CSF antibody titers the AI has the advantage to verify an intrathecal antibody synthesis and allows to quantify this synthesis. Sensitivity of testing for anti-NMDAR-IgG is higher in CSF than in serum^5^. Furthermore, anti-NMDAR antibodies in serum, especially with low titers, have been reported in patients without anti-NMDAR encephalitis or immune-mediated disorders^21^. Interpretation of cell-based assays (CBA) requires experience, and an unspecific staining raises the risk of false-positive results^22^. As well, in a small subset of patients (11/2600; 0,4%) CSF antibodies detected by CBA were not associated with anti-NMDAR encephalitis but with other neuroinflammatory (6/11) or non-inflammatory diseases^7^. Higher anti-NMDAR-IgG CSF and serum titers were described in anti-NMDAR encephalitis patients with poor outcome^5,23^. In addition, a weak correlation of the mRS and Clinical Assessment Scale of Autoimmune Encephalitis (CASE) items corresponding to psychiatric symptoms with CSF anti-NMDAR antibody titers was recently described^21^. We detected higher anti-NMDAR-IgG antibody titers in the CSF and NMDAR AI in patients fulfilling the Graus criteria of anti-NMDAR encephalitis in comparison with anti-NMDAR antibody positive patients who did not meet these criteria. In serum no differences in antibody titers were present. Additionally, a higher disease severity reflected by the mRS at time of sample collection correlated with the magnitude of anti-NMDAR-IgG titer in CSF. This is in line with a previous observation that quantitative intrathecal immunoglobulin synthesis was moderately higher in anti-NMDAR encephalitis with mRS score ≥ 3^4^. Even so the NMDAR AI at disease onset did not correlate with the mRS. A higher NMDAR AI was associated with a more pronounced clinical improvement measured via the delta mRS indicating a preferable treatment response if a high intrathecal antibody production is present. This matches the overall observations of a weak correlation between the level of anti-NMDAR-IgG in the CSF and disease severity and disease course. Yet, the pathogenicity of anti-NMDAR-IgG has been proven by internalization of the receptor after antigen binding, the antibody titers and the AI might insufficiently reflect the complex processes in the CNS in this disease. This is also supported by the absence of a correlation between the interindividual AI- and mRS- course in our current study (delta AI vs. delta mRS: rs = 0.1, p = 0.75). A hypothetical confounding effect might be explained by a theory that anti-NMDAR antibodies are absorbed in the brain hampering their detection in cerebrospinal fluid^24^. However, this theory has not been proven in humans.

Nevertheless, a negative AI had a high negative predictive value for the diagnosis of definite anti-NMDAR encephalitis. In our study population none of the patients with an anti-NMDAR encephalitis fulfilling the Graus diagnostic criteria for *definite anti-NMDAR encephalitis* had an NMDAR AI lower than two. The specificity for a positive NMDAR AI anyhow was low (0.38). This result suggests that intrathecal production of anti-NMDAR antibodies is necessary in anti-NMDAR encephalitis. The NMDAR AI might therefore be a valuable diagnostic tool to support differentiation between patients with an anti-NMDAR encephalitis and a positive antibody result of unknown significance. This corroborates the observation that in anti-NMDAR encephalitis the NMDAR AI can aid to obtain the correct differential diagnosis^25^ but extends this knowledge by adding that a positive NMDAR AI, demonstrating an intrathecal production of anti-NMDAR-IgG antibodies, has a low specificity for the diagnosis of anti-NMDAR encephalitis. This improves our current understanding of anti-NMDAR encephalitis and furthermore provides a possible aid to the clinical routine for the diagnosis of anti-NMDAR antibody encephalitis. Hence, the calculation of a NMDAR AI can be particularly useful in cases of atypical initial manifestations, such as unusual psychiatric symptoms. In these cases, where the AI is less than or equal to 2, our results suggest that the likelihood of anti-NMDAR encephalitis is low and this may prevent ineffective and potentially harmful treatments. Therefore, the NMDAR AI serves as a unique tool within the current diagnostic possibilities for identifying potentially false-positive anti-NMDAR encephalitis diagnoses. Another advantage is that no further obstacles need to be overcome before implementing this approach in clinical practice, as CSF and serum analyses, traditionally employed in diagnosing anti-NMDAR encephalitis, already provide the necessary data for calculating the NMDAR AI.

The strength of this study is that it is a large monocenter study including 42 patients with anti-NMDAR-IgG antibodies. Antibody titers were titrated manually, and end-point titer levels were carefully assessed in a blinded manner to the subgroup attribution.

However, some limitations need to be addressed. Due to the retrospective analysis, outcome measures were restricted. Therefore, we could not assess outcome parameters like the Clinical Assessment Scale in Autoimmune Encephalitis (CASE) in addition to mRS. Follow-up data were available for 17 patients, limiting conclusions on outcome measurements, probably explaining the missing correlation of NMDAR AI to the NEOS score. The NMDAR AI can only be calculated in patients with positive anti-NMDAR antibodies in serum and CSF, restricting its use to these patients. The results should be verified in larger study populations, yet in a multicenter approach interrater reliability of anti-NMDAR titers needs to be carefully addressed.

In conclusion, these results allow to draw the following clinically relevant conclusions: (a) negative AI for anti-NMDAR antibodies has a high negative predictive value for the diagnosis of anti-NMDAR encephalitis, (b) a positive NMDAR AI alone does not allow the diagnosis of anti-NMDAR encephalitis, (c) higher CSF antibody titers are correlated with disease severity (d) a higher NMDAR AI at disease onset is associated with an improved clinical outcome.

### Supplementary Information

Supplementary Information 1.
