# Supplementary figures and images for: The relevance of NMDA receptor antibody-specific index for diagnosis and prognosis in patients with anti-NMDA receptor encephalitis
Source: Sci Rep. 2023 Aug 4;13:12696. doi: 10.1038/s41598-023-38462-6 (PMC10403579; doi:10.1038/s41598-023-38462-6)

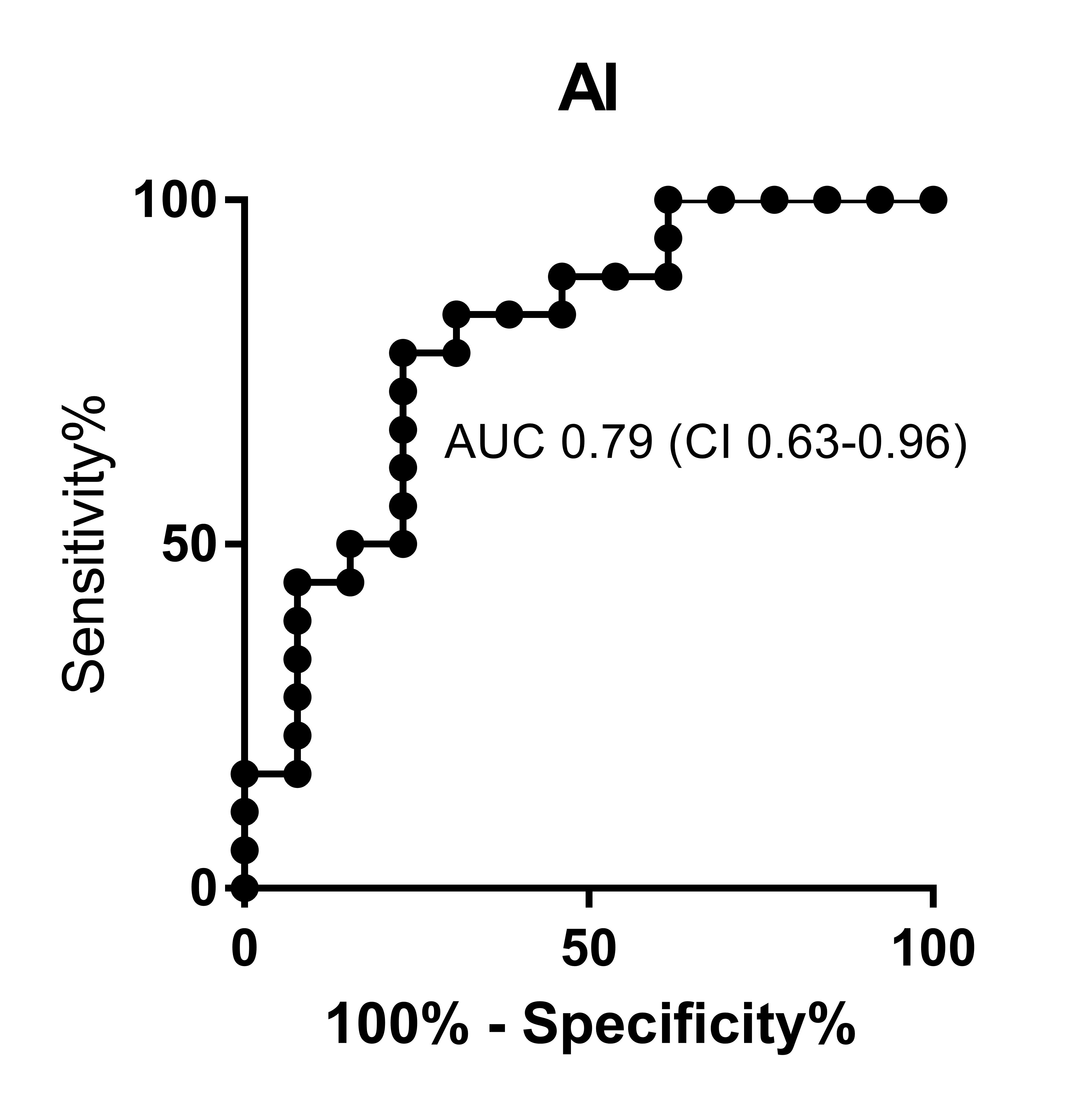

Supplement: Supplementary file 1 — Supplementary Information 1. [file 41598_2023_38462_MOESM1_ESM.jpg]
